# The Use of Developmental Rehabilitation Services. Comparison between Bedouins and Jews in the South of Israel

**DOI:** 10.1100/tsw.2004.18

**Published:** 2004-03-15

**Authors:** Hasia Lubetzky, Shifra Shvarts, Joav Merrick, Gideon Vardi, Aharon Galil

**Affiliations:** ^1^ Zusman Child Development Center, Division of Pediatrics, Soroka University Medical Center, Faculty of Health Sciences, Ben Gurion University of the Negev, Beer-Sheva, Israel; ^2^ Department of Health Systems Management, Division of Public Health Sciences, Faculty of Health Sciences and School of Management, Ben Gurion University of the Negev, Beer- Sheva, Israel; ^3^ National Institute of Child Health and Human Development, Office of the Medical Director, Division for Mental Retardation, Ministry of Social Affairs, Jerusalem, Israel

**Keywords:** child health, human development, public health, disability, rehabilitation services, Bedouin, Jews, Israel

## Abstract

Some communities have peripheral zones inhabited by persons with a different culture than the majority of the general population, such as the Aboriginals in Australia, the Native Americans in the U.S. and Canada, the Eskimos in Lapland, and the Bedouins in Israel. These citizens are not receiving the same medical or rehabilitation services as the citizens of the metropolitan areas due to the fact that health and welfare programs are not adapted to their unique needs. At the Soroka University Medical Center in Beer-Sheva, Israel, the health and rehabilitation services have a very large and heterogeneous catch-up population serving most of the south of Israel. The purpose of this study was to look at the utilization and the number of appointments for child rehabilitation services by the Bedouin population compared to the general population in the south of Israel at the Zusman Child Development Center (CDC).The records of appointments to the CDC between the years 1995—1999 inclusive were studied and we randomly chose to limit the study to January, April, July, and October of each year, and randomly chose the daily records of nine therapists, three from each discipline (occuptional therapy [OT], physical therapy [PT], and speech and language therapy [SLT]). There were 8,504 appointments during these 4 months of the years 1995—1999, 2,255 of which were for Bedouin and 6,249 for Jewish children. Noncompliance with therapy appointments (NCTA) for the same period for both the Bedouins (31%) and Jewish children (26%), with a significant difference between the two populations, was noted. Of all the Jewish childrens appointments, the percentage of all three services was similar: 33% to PT, 38% to OT, and 29% to SLT, but for the Bedouin children, the percentage between the three services was significantly different: 62% to PT, 34% to OT, and 3% to SLT. These results seem to indicate that the Bedouin families prefer the PT and OT over the SLT. Our results enhanced the need for planning a model for supplying health services adapted to clients coming from different cultures. According to this model, we need to take into consideration the cultural differences, the accessibility to rehabilitation services, and the economical impact on the family; all in all, to give a better solution to the patient with special needs.

## INTRODUCTION

The health services provided to insured citizens — in countries like Scandinavia, Australia, New Zealand, Canada, and the U.S. — are among the most progressive in the world. There are also many laws defining the rights of the handicapped or disabled with the obligations of the government. Such rights emerged from the concept that all citizens, including the disabled, deserve welfare from the government and society as a whole has responsibility.

There are some communities in the peripheral zones of several places who have a different culture from the majority of the population, such as the Aboriginals in Australia, the Native Americans in the U.S. and Canada, the Eskimos in Lapland, and the Bedouins in Israel. These citizens are not receiving the same medical or rehabilitation services as the citizens of the metropolitan areas due to the fact that health and welfare programs are not adapted to their unique needs[1,2,3].

Apparently, everyone approaches the health services when in need of them, but according to Helman[4], people use these services only when they are within reach, within their financial capacity, and within cultural acceptance.

At the Soroka University Medical Center in Beer-Sheva, Israel, the health and rehabilitation services have a very large and heterogeneous catch-up population serving most of the south of Israel. The citizens come from many ethnic, cultural, and religious groups. The two main groups are Jews, who are the majority in Israel, and the Bedouins, who are about 20% of the citizens in the south region[5]. Most of the Jews using the rehabilitation services come from the cities and major villages in the south, while in some communities, the citizens use local facilities and the connection with the rehabilitation center is for instruction and follow-up of children with multiple and complex disabilities.

The Bedouins of the Negev are in transition from a Nomadic life to more permanent settlements. They are an ethnic, religious, and cultural minority with many socioeconomical problems. Their language is Arabic, and usually only the men are able to speak Hebrew. Many of the men are unemployed and their families live on social security income. The medical and rehabilitation services offered by the health authorities is not fully used due to language, cultural, and economic difficulties, but also due to the geographical scattering[5,6,7].

The meeting between the Jewish therapist and the Bedouin patient is a meeting between two different cultures who appraise the value of health and rehabilitation in different ways. This clash of cultures is also well known in other western countries, where health services providers stem from the medium-high socioeconomic group — educated in western medicine based on scientific logic and assumptions that must be examined by firm and objective criteria — who meet patients from totally different cultures[8,9].

## The Zusman Child Development Center (CDC) at Soroka University Medical Center

At the Soroka University Medical Center, there are 12,000 newborns every year with close to 50% Bedouins, even though they only constitute 20% of the population in the south. More than 80% of the children with any developmental problem are referred to the CDC. About 5% (350 every year) are diagnosed with moderate or severe disabilities. About 10% have a mild disability with motor, communication, or learning difficulties.

Every year, a total of about 750 new children are referred to the CDC for assessment and treatment by the medical and paramedical staff. About 50% need treatment, 30% need follow-up, and 20% are discharged without need for further intervention. After diagnosis, the therapist and the parents plan an integrative program with special neurological and developmental components suitable for the child, the family, and the rehabilitation services in the local community where the child lives. The rehabilitation therapy is given two to four times a month. The frequency of treatments and follow-up depends on the severity of the disability, the capacity of the family to come to the therapist, and the possibilities for improvement.

The therapist plans the treatment at home and during the session, the parents receive instructions connected to the development of the child with a view to capacities and limitations.

The purpose of this study was to look at the utilization and the number of appointments for rehabilitation services by the Bedouin population compared to the Jewish population in the south of Israel.

## METHODS

The degree of utilization of services was evaluated by two parameters:

1. Comparison of the ratio of appointments between the Bedouin and Jewish population to the three rehabilitation services in the CDC: occupational therapy (OT), physiotherapy (PT), and speech and language therapy (SLT).
2. Comparison of the rate of "not attending" predetermined appointments or noncompliance with therapy appointments (NCTA) between the two populations.

### The Study Population

The pediatric population of the southern part of Israel receives medical care and developmental follow-up and therapy at the regional area hospital, the Soroka University Medical Center in Beer-Sheva. The study is based on cluster samples. We reviewed the records of appointments to the CDC, medical, psychosocial, and allied professions staff between the years 1995–1999 inclusive. We randomly chose to limit the study to January, April, July, and October of each year, and we randomly chose the daily records of nine therapists, three from each discipline (OT, PT, and SLT).

There were 8,504 appointments during these 4 months of the years 1995–1999, 2,255 of which were for Bedouin and 6,249 for Jewish children. The dependent variable was the percentage of NCTA from the two populations.

### Statistical Analysis

The raw data were analyzed using SPSS (version 10) and nonparamedic comparisons ($\chi^2$) of variables between the two populations; statistical significance was determined by a $p < 0.05$).

## RESULTS

In Table 1, the total number of appointments for the sample during the 5 years is shown together with the NCTA for the same period for both the Bedouin and Jewish children with a significant difference between the two populations.

**TABLE 1**
**Number of NCTA in Bedouins and Jews at the Zusman CDC in Beer-Sheva During the Years 1995–1999 (*p* < 0.001)**

| | Jews | Bedouins |
|---|---|---|
| Total number of appointments | 6,249 | 2,255 |
| NCTA | 1,624 | 702 |
| Percentage of NCTA | 26% | 31% |

In Table 2, the ratio between the appointments of Bedouins and Jews is presented for the three different rehabilitation services (OT, PT, and SLT) and in Fig. 1, the percentage of appointments of Bedouin and Jewish children to the three different rehabilitation services is shown. The data showed a significant statistical difference between appointments of Jewish children compared to Bedouin children regarding PT to OT, regarding PT to SLT, and regarding OT to SLT. Of all the Jewish childrens' appointments, the percentage of all three services was similar: 33% to PT, 38% to OT, and 29% to SLT, but for the Bedouin children, the percentage between the three services was significantly different: 62% to PT, 34% to OT, and 3% to SLT. These results seem to indicate that the Bedouin families prefer the PT and OT over the SLT. These preferences are expressed both in the numbers of appointments given to the Bedouins to PT as for OT and SLT (1,410, 769, 76, respectively, as shown in Table 2) and in the ratio of appointments between Jews and Bedouins to each of the three services: PT compared to OT and compared to SLT (1.4-, 3-, and 23.4-fold, respectively, as shown in Fig. 1).

**TABLE 2**
**The Ratio between the Appointments of Bedouin and Jewish Children at the Zusman CDC to the Three Different Rehabilitation Services during the years 1995–1999 (p < 0.01)**

| | Jew's Appointments | Bedouin's Appointments | Ratio Jews to Bedouins |
|---|---|---|---|
| PT | 2,016 | 1,410 | 2,016/1,410 = 1.43 |
| OT | 2,326 | 769 | 2,326/769=3.02 |
| PT | 2,016 | 1,410 | 2,016/1,410 = 1.43 |
| SLT | 1,817 | 76 | 1,817/76 = 23.9 |
| OT | 2,326 | 769 | 2,326/769 = 3.02 |
| SLT | 1,817 | 76 | 1,817/76 = 23.9 |

## DISCUSSION

The first preliminary assumption that the total NCTA rates to the rehabilitation services would be higher in the Bedouins comparing to the Jews proved to be correct. The NCTA rates were significantly higher compared to the NCTA rates in Jewish children over all 5 years.

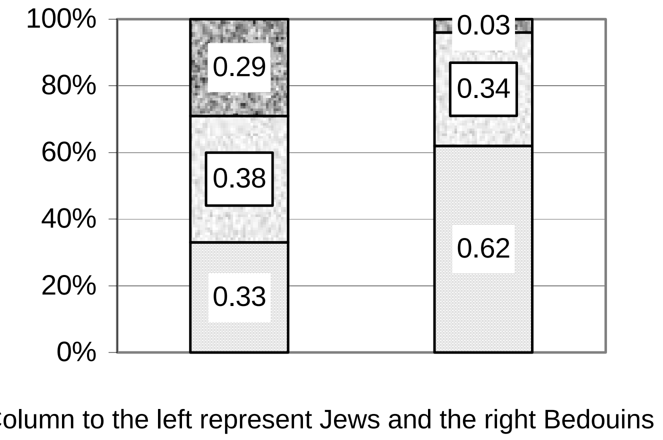

**FIGURE 1.** The percentage of appointments to the three rehabilitation services (PT = brick, OT = gray, SLT = black area) at the Zusman CDC, 1955–1999.

The rehabilitation services at the CDC are based on western values and not adapted to the cultural values and life style of the Bedouins. They are also less available to the Bedouins living at a distance from the medical center. These factors could explain why the Bedouins use the rehabilitation services to a much lesser extent than the Jews[5,10].

These findings strengthen the claim by Saha et al.[8] and Flores[9] that the use of health services is influenced by the adaptation of the treatment to the unique needs of the patient. According to this perception, utilization by the individual is dependant on the user's view about health, morbidity, and the treatment he or she gets. Culture and lifestyle are the main factors influencing these perceptions[11]. These results are similar to those found by Galil et al.[5], where the compliance to rehabilitation therapy was lower in the Bedouin than the Jewish population. It was also found in that study that the Bedouins believe in fate as a very potential factor in the birth of a child with development problems. They have less trust in the capacity of the western rehabilitation system to help their disabled child. The cultural influence on the use of rehabilitation services depended not only on the cultural differences, but also on the perception of the importance of the rehabilitation therapy to make a difference for the disabled child.

These results are also in agreement with the findings of Jones et al.[12] and Strasser[1] concerning the influence of accessibility on the use of health services. The further the patients live from the medical center, the greater the indirect expenses and the dropout from treatment compared to the patient living closer to the urban medical center.

The second assumption we made, that the rates of appointments will be higher in the Jews, also proved to be correct. Analyzing the data in Table 2, the difference between the low number of Bedouin appointments to SLT services (N = 76) compared to the high number of Jewish appointments (N = 1,817) can clearly be seen.

When comparing the appointment rate between Bedouins and Jews to the three rehabilitation services, we found a significant difference ($p < 0.01$) between the appointment rate of Jews vs. Bedouins comparing PT/OT, PT/SLT, and OT/SLT and the number of Bedouin appointments to P/T (1,410) was higher than their appointments to SLT appointments (76). We can explain the small amount of appointments to SLT vs. PT by the cultural influence on the use of rehabilitation services. It seems that the lesser use of SLT services at the CDC could be due to the fact that the Hebrew language is the main therapy tool of this sector. The Hebrew language is not adapted to the cultural world of the Bedouin child and not responsive to his needs. The emphasis of SLT treatment is how to deal with problems arising from daily needs of using language and communicating skills, which is influenced by cultural values. The great difficulty to promote the Bedouin child without a common language is another reason for the low number of appointments[8].

A possible explanation for the major Bedouin use of PT is the great importance in the Bedouin culture to the issue of movement and walking. The parenting culture encourages the upright position from the age of 4 months. The explanation given by the parents is that a baby crawling in the tent or shed is more vulnerable to be damaged by open fire or harmful insects.

The differences exposed within the use of various rehabilitation services at the CDC enforced the statement by Helman[4] about the influence of culture on individual perception of offered therapy and the claim of Jones et al.[12] about the influence of accessibility on the use of health services. These factors cause frustration in both sides, disrupting the therapeutic bonds and not allowing the patient to continue with long-term treatment and follow-up.

## CONCLUSIONS

In this research, we found a lesser use of developmental rehabilitation services in southern Israel by the Bedouins, which can be explained by a different culture and lesser accessibility to rehabilitating services.

Physicians and allied professionals familiar with factors affecting use of rehabilitation services by different populations will be better able to diagnose, display appropriate attitudes, and improve treatment to the patient, so that compliance and persistence of therapy will improve.

During the last few years, a cultural pointed therapy had been developed. According to this attitude, the staff members are getting acquainted with the social, economical, and cultural aspects of how health and illness are perceived by the patient within his cultural framework and not the cultural framework of health providers[11]. The cultural pointed therapy is not in place of the traditional therapy, but a supplemental therapy.

Our results enhanced the need for planning a model for supplying health services adapted to clients coming from different cultures. According to this model, we need to take into consideration the cultural differences, the accessibility to rehabilitation services, and the economical impact on the family; all in all, to give a better solution to the patient with special needs.

## BIOSKETCHES

**Hasia Lubetzky, OT, MHA,** is a Director of Occupational Therapy Services, Soroka University and an occupational therapist at the Zusman. She is currently working on her PhD thesis "Supply of Health Services to Rural Areas. A Suggested Model for Israel" in the Department of Health Systems Management, Division of Public Health Sciences, Faculty of Health Sciences and School of Management, Ben Gurion University of the Negev. **E-mail** hasia@bgumail.bgu.ac.il or hasil@clalit.org.il

**Shifra Shvarts, PhD,** is Professor and Head of the Department of Health Systems Management, Division of Public Health Sciences, Faculty of Health Sciences and School of Management, Ben Gurion University of the Negev, Beer-Sheva, Israel. With a BA in history and geography, an MA in history, and a PhD in health sciences, her interests and numerous publications center around history of medicine and public health, development of the healthcare system in Israel, development of health organizations, health insurance legislation, and the development and history of maternal and infant welfare centers. E-mail: shvarts@bgumail.bgu.ac.il, Website: http://fohs.bgu.ac.il/people/PDetails.asp?StaffID=11733

**Aharon Galil, MD,** is Associate Professor of Pediatrics and the Director of the Zusman Child Development Center, Division of Pediatrics, Faculty of Health Sciences, Ben Gurion University of the Negev, Beer-Sheva, Israel. Dr. Galil has numerous publications in the field of child health and human development, rehabilitation, intellectual disability, and disability. E-mail: galil@bgumail.bgu.ac.il

**Joav Merrick, MD, DMSc,** is Professor of Child Health and Human Development affiliated with the Zusman Child Development Center, Division of Pediatrics and Community Health at the Ben Gurion University of the Negev, Beer-Sheva, Israel; the Medical Director of the Division for Mental Retardation, Ministry of Social Affairs, Jerusalem; and the Founder and Director of the National Institute of Child Health and Human Development. He has numerous publications in the field of child and human development, rehabilitation, intellectual disability, disability, health, welfare, abuse, advocacy, quality of life, and prevention. Dr. Merrick received the Peter Sabroe Child Award for outstanding work on behalf of Danish Children in 1985 and the International LEGO-Prize ("The Children's Nobel Prize") for an extraordinary contribution towards improvement in child welfare and well being in 1987. E-mail: jmerrick@internet-zahav.net. Website: www.nichd-israel.com

**Gideon Vardi, MD, MPH,** is a specialist in pediatrics, child health and development working at the Zusman Child Development Center, Division of Pediatrics, Faculty of Health Sciences, Ben Gurion University of the Negev, Beer-Sheva, Israel. Dr. Vardi's interest and several publications are in the field of child health and human development, rehabilitation, intellectual disability, and disability. E-mail: GideonVa@clalit.org.il
